# Feeding problems, age of introduction of complementary food and autism symptom in children with autism spectrum disorder

**DOI:** 10.3389/fped.2022.860947

**Published:** 2022-08-12

**Authors:** Tiantian Wang, Junyan Feng, Yang Xue, Ling Shan, Feiyong Jia, Xiaojing Yue

**Affiliations:** Department of Developmental and Behavioral Pediatrics, The First Hospital of Jilin University, Jilin University, Changchun, China

**Keywords:** autism, children, complementary foods, age, feeding problem

## Abstract

In this cross-sectional study, 84 children with autism spectrum disorder (ASD) and 77 healthy subjects showing typical development (TD) were reviewed. Parents reviewed the age of introduction of complementary foods (CFs), completed a demographic, diet behavior questionnaire and the Autism Behavior Checklist (ABC). The results showed that the age of introduction of CFs was later in children with ASD than their TD counterparts. The age of introduction of CFs in ASD group was positively correlated with feeding problem. While the correlation was not observed in TD group. Children in the ASD group had higher total scores of the diet behavior questionnaire and all four subdomains (poor eating ability, mealtime eating behavior, food selectivity, and parental feeding behavior). ASD symptoms were clearly associated with feeding problems. The sensory subdomain score in ABC was positively correlated with poor eating ability, mealtime behavior and total score of the diet behavior questionnaire. The social self-care subdomain score was positively correlated with food selectivity. The interaction subdomain score was negative correlated with parental feeding behavior and total score of the diet behavior questionnaire. Further studies are required to establish the utility of delayed CFs introduction and/or early feeding problems as potential indicators of ASD.

## Introduction

Autism spectrum disorder (ASD) is a heterogeneous neurological developmental disorder typically characterized by difficulties in communication and socialization, frequently presenting traits including fewer interests, abnormal sensory processing, and repetitive behaviors (1). The prevalence of ASD is 1 in 44 in 8-year-old children with an estimated male–female ratio of 4.2:1 (2). Feeding and eating problems are prevalent in children with ASD with a range from 40.3 to 96% (3–6). Food selectivity, food refusal and poor eating behaviors are prevalent in children with ASD (7–9).

Eating is a primary function in the human development. The period of early childhood is key in nutrition. Especially during the infancy phase, the children learn to ingest the fluid and solid food. It is not only crucial to the nutritional programming or metabolic programming, but also important to intellectual potential and physical fitness. The children should learn to ingest specific food and accept new tastes at specific age. Disruption of the process of learning to eat and accept new tastes during the critical “window” of opportunity can result in both oral-sensory and oral-motor dysfunction (10). The physical process of feeding can be disrupted through neurodevelopmental disabilities.

At present, the researches on feeding problem of ASD children mostly focus on children over 3 years old. Research on early feeding disorders in ASD has been limited. Brzóska et al. (11) highlighted that feeding problems might show up very early in the course of ASD, and dietary problems are more common during the 1st year of life from the time of introduction of complementary foods (CFs). Emond et al. (12) reported late introduction of solid foods in infants with ASD after 6 months, described as “slow feeders” at 6 months. Owing to the prevalence of feeding problems in ASD children and associated negative consequences, clinicians should be alert to the presence of these symptoms early in life. However, limited empirical information is available on the age of introduction of CFs in ASD infants. The current study was conducted to determine the potential interrelationships between age of introduction of CFs, feeding problems and ASD symptoms. A comparison group of children showing typical development (TD) was included to assess the differences in feeding problems and age of introduction of CFs relative to subjects with ASD. The main aims of the study were to: (1) compare the age of introduction of CFs between children with and without ASD, (2) compare feeding problems between children with and without ASD, and (3) examine the relationships between age of introduction of CFs, feeding problems and symptoms of ASD.

## Methods

### Participants

This was a cross-sectional study of children with ASD (*N* = 84) and healthy controls (*N* = 77). The data were collected from clinical notes of children between the ages of 2 and 5 who were diagnosed with ASD in the Department of Developmental and Behavioral Pediatrics (The First Hospital of Jilin University, Changchun, China) from October 2018 to April 2019. They were diagnosed by a multidisciplinary team following the American Psychiatric Association Diagnostic and Statistical Manual of Mental Disorders (DSM)-5 criteria. Regarding ASD severity level, 42 children (50%) had mild ASD, 33 children (39%) had moderate ASD, 9 children (11%) had severe ASD. Seventy-nine children (94%) had coexisting global developmental delay. The data of healthy controls that were collected from the clinical notes of the healthy children who were routine check-ups in the same hospital during the same time. Children with a history of other neurological and severe somatic disease, head trauma and uncontrolled seizures were excluded from study. The study protocol was approved by the ethics committee of the First Hospital of Jilin University and informed consent obtained from all parents.

### Measures

Parents completed a demographic questionnaire on general information (age, gender, date of birth, birth weight, BMI, early feeding pattern, parents' educational level, family income and age of introduction of CFs). Parents also completed a diet behavior questionnaire developed by Zhou et al. based on the Children's Eating Behavior Inventory (CEBI), Mealtime Behavior Questionnaire (MBQ), Children's Eating Behavior Questionnaire (CEBQ), Chinese version of the Identification and Management of Feeding Difficulties (IMFED), and adjusted according to Chinese dietary habits (13). Cronbach's alpha coefficient with the diet behavior questionnaire of Zhou and co-workers was 0.86, suggesting robust internal consistency (13). The questionnaire consists of 29 items and measures four main factors: poor eating ability, mealtime eating behavior, food selectivity, and parental feeding behavior. The poor eating ability scale mainly measures whether children have difficulties in eating, such as mastication or swallowing. Mealtime behavior mainly measures children's bad behavior when eating, such as leaving the table. Food selectivity measures whether children have food choices, such as eating the same food repeatedly or rejecting a certain food. Parental feeding behavior measures parents' feeding behavior, such as chasing their children to feed, or always worrying about their children eating less. A 5-point Likert scale was used. For each item, frequency was assessed based on response options of: never (0), rarely (1), sometimes (2), often (3), and always or almost always (4). The Autism Behavior Checklist (ABC) was applied to assess ASD symptoms. The ABC is a list of 57 “yes” or “no” questions, with each question corresponding to a specific symptomatic category whereby five categories are evaluated: sensory, social and self-help, interaction, stereotypes and object use, and language. The scores of single items are rated 1 to 4, with a total score of ≥53 used as a cutoff value for suspected autism (14).

### Statistical analysis

SPSS23.0 software was used for statistical analysis of data. Continuous variables with normal distributions are represented as the means ± standard deviations (SDs) and the two independent samples *t*-test used for comparison between groups. Continuous variables with abnormal distributions are represented as the medians (P25–P75) and the Mann-Whitney U test used for comparison between groups. Categorical variables are represented as frequencies (percentages) and the χ2 test used for comparison between groups. Correlation tests were performed using Spearman's correlation analysis. Differences were considered statistically significant at *P* < 0.05.

To manage possible differences between the two groups, we used generalized linear model (GLM) for further group comparisons to minimize the impact of other factors. We used the age of introduction of CFs and the scores of the diet behavior questionnaire as dependent variables, respectively, included categorical (group, sex) on which the two groups differed significantly as factors and covariates, respectively and ran the regression models.

## Results

### Comparison of clinical baseline data between the ASD and TD groups

The study included 84 children with ASD and 77 TD children. No significant differences in chronological age, body mass index (BMI), birth weight, feeding patterns, family income, and degree of maternal and paternal education were observed between ASD and TD groups. The ASD group contained more boys than the TD group (83.3 and 59.7%, respectively, *p* = 0.001) (Table 1).

**Table 1:** Demographic data of TD and ASD subjects.

	**TD (*N* = 77)**	**ASD (*N* = 84)**	***t/χ^2^/U***	***p***
Gender			11.104	0.001*
Male	46 (59.7%)	70 (83.3%)		
Female	31 (40.3%)	14 (16.7%)		
Age (month)	35 (28–44)	33 (28–41)	−0.962	0.364
BMI (kg/m^2^)	17.03 ± 1.90	17.01 ± 2.50	0.048	0.962
Birth weight (kg)	3.46 ± 0.49	3.46 ± 0.58	−0.063	0.95
Feeding pattern			1.705	0.192
BF	21 (27.3%)	31 (36.9%)		
FF	56 (72.7%)	53 (63.1%)		
Maternal education			0.023	0.879
Primary	44 (57.1%)	49 (58.3%)		
High	33 (42.9%)	35 (41.7%)		
Paternal education			0.287	0.592
Primary	49 (63.6%)	49 (59.5%)		
High	28 (36.4%)	35 (40.5%)		
Family income (RMB)	5000 (3333–6667)	6667 (3333–8333)	−1.652	0.099

BF, Breastfeeding; FF, formula feeding; TD, typical development; ASD, autism spectrum disorder; CFs, complementary feeding, *p < 0.05.

### The age of introduction of CFs in the ASD and TD groups

We performed GLM in order to account for the significant baseline differences in our groups as we examined the age of introduction of CFs. For these analyses we used the age of introduction of CFs as dependent variables. The sex which the groups significantly differed at baseline, was included as covariates when analyzing group difference. The sex has no significant effect on the age of introduction of CFs (*P* > 0.05). The age of introduction of CFs of the TD group were lower than those of the ASD group (B = −0.740, *P* < 0.05) (Table 2).

**Table 2:** Comparison of the age of introduction of CFs in the ASD and TD groups.

	***B***	**SE**	**Waldχ^2^**	***p***
**Age of CFs**
TD group	−0.740	0.2824	6.872	0.009*
ASD group	-	-	-	-
Sex (Male)	0.257	0.3144	0.666	0.414
Sex (Female)	-	-	-	-

ASD, autism spectrum disorder; TD, typical development; CFs, complementary foods, *p < 0.05.

### Eating and feeding problems between ASD and TD groups

To address the issue of group differences in eating and feeding problems, we examined the scores obtained with the diet behavior questionnaire. We performed GLM in order to account for the significant baseline differences in our groups as we examined the scores obtained with the diet behavior questionnaire. For these analyses we used the scores obtained with the diet behavior questionnaire as dependent variables. The sex which the groups significantly differed at baseline, was included as covariates when analyzing group difference. After adjusting for the effect of baseline sex, total scores of the TD group were lower than those of the ASD group (*B* = −8.178, *P* < 0.05). Furthermore, subjects in the TD group had significantly lower scores for all four subdomains (poor eating ability, mealtime behavior, food selectivity, and parental feeding behavior) compared to those in the ASD group (*P* < 0.05; Table 3).

**Table 3:** Comparison of feeding problems between ASD and TD groups.

		***B***	**SE**	**Waldχ^2^**	***p***
PEA					
	TD group	−1.750	0.4531	14.913	<0.0001*
	ASD group	-	-	-	-
	Sex (Male)	−0.829	0.5044	2.704	0.1
	Sex (Female)	-	-	-	-
MB					
	TD group	−2.453	0.8198	8.593	0.003*
	ASD group	-	-	-	-
	Sex (Male)	−2.388	0.9126	6.848	0.009*
	Sex (Female)	-	-	-	-
FS					
	TD group	−1.910	0.6082	9.864	0.002*
	ASD group	-	-	-	-
	Sex (Male)	−1.381	0.6770	4.161	0.041*
	Sex (Female)	-	-	-	-
PFB					
	TD group	−2.270	0.6163	13.564	<0.0001*
	ASD group	-	-	-	-
	Sex (Male)	−1.419	0.6861	4.280	0.039*
	Sex (Female)	-	-	-	-
Total					
	TD group	−8.178	1.9271	18.008	<0.0001*
	ASD group	-	-	-	-
	Sex (Male)	−6.291	2.1451	8.600	0.003*
	Sex (Female)	-	-	-	-

PEA, poor eating ability; MB, mealtime behavior; FS, food selectivity; PFB, parental feeding behavior, *p < 0.05.

### Relationships between age of introduction of CFs and eating and feeding problems in subjects of TD and ASD group

No statistically significant correlation was observed between the age of introduction of CFs and eating and feeding problems in the TD group (*P* > 0.5). The age of introduction of CFs was positively correlated with the total diet behavior questionnaire score in the ASD group. Correlation analysis was further performed to determine the association between age of introduction of CFs and the four subdomains of the diet behavior questionnaire in the ASD group. Statistically significant correlation was observed only for mealtime behavior. Specifically, the age of introduction of CFs was positively correlated with the score for mealtime behavior (Table 4).

**Table 4:** Relationships between age of introduction of CFs and eating and feeding problems in subjects of TD and ASD group.

		**PEA**	**MB**	**FS**	**PFB**	**Total**
TD	r	−0.113	−0.005	0.035	−0.01	−0.031
	*p*	0.329	0.965	0.761	0.93	0.791
ASD	r	0.171	0.328	0.113	0.098	0.25
	*p*	0.119	0.002*	0.307	0.374	0.022*

PEA, poor eating ability; MB, mealtime behavior; FS, food selectivity; PFB, parental feeding behavior, ^*^p < 0.05.

### Relationships between age of introduction of CFs and symptoms in subjects with ASD

Correlation analysis between the age of introduction of CFs and ABC scores used to assess ASD symptoms was performed in the ASD group. No significant associations were identified, either for total ABC scores or scores of the five subdomains (sensory, social and self-help, interaction, stereotypes and object use, and language; Table 5).

**Table 5:** Relationships between age of introduction of CFs and symptoms in subjects with ASD.

**rs**	**Total**	**BU**	**Sensory**	**SS**	**Language**	**Interaction**
*r*	−0.011	−0.011	0.002	−0.017	−0.137	0.009
*p*	0.92	0.92	0.986	0.876	0.215	0.937

BU, Body and object use; SS, social and self-help.

### Relationships between eating and feeding problems and symptoms in subjects with ASD

Correlation analysis was further performed for the associations between five subdomains of ABC and four subdomains of the diet behavior questionnaire in the ASD group. The sensory subdomain score was positively correlated with poor eating ability, mealtime behavior, and total score of the diet behavior questionnaire. No significant correlation was found for food selectivity and parental feeding behavior. The social and self-help subdomain score was positively correlated with food selectivity. No significant correlation was found for poor eating ability, mealtime behavior, parental feeding behavior and total score. The interaction subdomain score was negatively correlated with parental feeding behavior and total score of the diet behavior questionnaire. No significant correlation was found for poor eating behavior, mealtime behavior and food selectivity. Body and object use subdomain, language subdomain and total ABC scores were not significantly correlated with scores of the total diet behavior questionnaire and its subdomains (Table 6).

**Table 6:** Relationships between eating and feeding problems and symptoms in subjects with ASD.

		**Total ABC**	**BU**	**Sensory**	**SS**	**Language**	**Interaction**
PEA	r	0.083	0.087	0.278	0.210	−0.044	−0.21
	*p*	0.453	0.431	0.01*	0.055	0.690	0.055
MB	r	0.050	0.117	0.217	0.037	0.062	−0.160
	*p*	0.649	0.289	0.047*	0.74	0.576	0.145
FS	r	0.054	0.028	0.212	0.223	0.010	−0.152
	*p*	0.627	0.8	0.053	0.041*	0.927	0.167
PFB	r	−0.117	0.002	0.171	0.067	−0.167	−0.344
	*p*	0.29	0.983	0.119	0.546	0.129	0.001*
total	r	0.007	0.091	0.274	0.132	−0.056	−0.288
	*p*	0.952	0.411	0.012*	0.233	0.613	0.008*

PEA, poor eating ability; MB: mealtime behavior; FS, food selectivity; PFB, parental feeding behavior; BU, Body and object use; SS, social and self-help, *p < 0.05.

## Discussion

In the present study, eating and feeding problems and age of introduction of CFs were compared between children with and without ASD. We additionally examined the potential associations between the age of CFs introduction, eating and feeding problems and ASD symptoms. The main findings were as follows: (1) compared to subjects with TD, most children with ASD were introduced to CFs at a later stage, (2) age of introduction of CFs was positively correlated with total score of the diet behavior questionnaire and mealtime behavior in the ASD group, and (3) children with ASD showed more feeding problems than the TD group, which were associated with symptoms of ASD.

Compared to children showing TD, those with ASD were introduced to CFs later, inconsistent with the report of Huxham et al. (15) showing that the mean age of introduction of CFs was earlier at 5 months in children with ASD. This difference may be due to the different definitions of CFs between the reports. Formula milk was not included as CFs in our study (16). Huxham et al. (15) examined infants who were exclusively breastfed and included formula in the definition of CFs. Their study showed that 38.8% subjects with ASD struggled to ingest spoon-fed pureed foods and 55.6% had difficulties with lumpy foods consistent with our findings. Zobel et al. (17) reported that ASD participants consumed pureed foods at 6.6 months relative to 5.89 months for TD participants, with no significant group mean differences. The group of Emond 2010 observed that children with ASD had difficulty in accepting solids after 6 months and proposed that this could be an early symptom of problems with accepting change by autistic subjects (12). Brzóska et al. (11) also found a delayed introduction of foods with solid and lumpy structure. These results also present delayed introduction of CFs consistent with our findings. The reason why children with ASD have delayed introduction of CFs we analyze may be the following aspects: (1) Introduction to CFs is one of the means by which infants interact with their parents. Responsive feeding is an important contributory factor to the success of introduction to CFs, emphasizing the interaction and emotional communication between parents and infants during feeding, which encourages infants to signal hunger and satiety and mothers to identify these cues and provide timely and appropriate responses (18). A core feature of ASD is social skill deficits. Poor emotional communication between parents and infants may make responsive feeding difficult, leading to delayed introduction of CFs. (2) In addition, children with ASD often show the typical sameness of routine, inflexibility, and fear of novelty, which may also be a potential reason for delayed introduction of CFs. The delay in acceptance of CFs in infants may be an early signal of ASD. (3) Moreover, the process of eating is associated with multiple sensations. Abnormalities in sensory processing processes to the environment are associated with ASD (19). Earlier reports suggest that infants diagnosed later with ASD are more perceptually sensitive to environmental stimuli (20–22). Infants with ASD may therefore be more sensitive to novel dietary experiences, leading to refusal to accept these foods and consequential delay in introduction of CFs in their diet.

We observed that the age of introduction of CFs was positively correlated with the total diet behavior questionnaire and eating behavior scores in the ASD group. While this correlation was not observed in TD group. Further investigation of the potential relationship between age of CFs introduction and symptoms of ASD (assessed via ABC scores) revealed no significant correlations. Delayed introduction of CFs may also be an early sign of eating and feeding problems in children with ASD.

In our study, children with ASD presented with more eating and feeding problems than their same-age non-ASD peers. Total scores along with scores for all four subdomains (poor eating ability, mealtime eating behavior, food selectivity, and parental feeding behavior) of children in the ASD group were consistently higher relative to the TD group, in keeping with empirical findings reported in the literature (17, 23–28). Further analysis of the correlation between feeding problems and autism symptoms showed that the sensory score was positively correlated with poor eating ability, mealtime behavior and total score of the diet behavior questionnaire. Our data on feeding problems support previous findings (8, 17, 19). Sensory impairments are frequent in children with ASD (29). Recent studies suggested that feeding problem in children with ASD may be related to sensory processing dysfunction (17, 19). Atypical sensory processing, such as sensitivity to color, taste, smell, and/or texture of food may lead children with ASD to refuse to food (30). Sensory impairments in children with ASD may be one factor that interferes with mealtime behavior (17). We did not detect a correlation between sensory score and food selectivity inconsistent with previous study, possibly since sensory behaviors were analyzed via subdomains of the ABC scale, which is not a professional sensory scale and incorporates a lower number of items for evaluation of food selectivity. However, we found the social self-care subdomain score was positively correlated with food selectivity. The reason may be children score higher in social self-care subdomain often cannot take food by themselves. Their parents may find more food selectivity problems when feeding them, compared with parents whose children can take food independently. However, some of our results were inconsistent with previous studies, which found no association between eating and feeding problems and interaction impairment severity (27). The interaction subdomain score was negatively correlated with parental feeding behavior and total score of the diet behavior questionnaire. One possibility to explain these associations is that children displaying better interactions respond to their parents more clearly that they do not want to eat the food their parents supply through expression, gesture or language, making it easier for parents to detect problems of their child. In this case, parents use more commands or distraction tactics by talking or coaxing the child to eat, which represent disruptive parental feeding behavior. Another reason maybe our sample size is small. This correlation may not exist in large sample studies because the correlation coefficient in our study is very small.

To our knowledge, this study is one of the first to report a significant association between age of introduction of CFs and feeding issues in children with ASD. The age of CFs introduction was positively associated with feeding problems in ASD group, not associated with feeding problems in TD group. The feeding problems were positively associated with symptoms of ASD. There may be some correlation between age of CFs introduction and symptoms of ASD. But our study did not find the correlation between age of CFs introduction and symptoms of ASD. It requires further investigation. Previous studies suggest that detection of early-life feeding problems is relevant for early diagnosis of ASD and could be potentially included as an ASD-specific screening tool (24). Our results may provide a preliminary point that the introduction of CFs can be included in detection of early-life feeding problems. Not just the time of introduction of CFs, but details of introduction of CFs. It requires further exploration. Feeding problems are additionally associated with symptoms of ASD. Accordingly, in clinical practice, the impact of ASD symptoms on feeding problems should be considered in comprehensive assessment and intervention approaches for children with ASD (6).

Our study has several limitations that should be taken into consideration. Firstly, the sample size was relatively small and differences in the male-female ratio were observed between the two sample groups. Since ASD is generally more prevalent in boys, our clinical sample was not equally distributed between the sexes. Although we made adjustments in statistics, future studies including the sex matched control group will be better. Secondly, we only analyzed the age of introduction of CFs and did not include more detailed information. Thirdly, our study was performed at a single center and involved a relatively small number of subjects. Further detailed multicenter, large-scale clinical studies on samples with well-matched sex ratios are warranted. In addition, we did not include parents' eating habits which also affect feeding. Further studies including factors of parents' eating habits are needed.

## Conclusion

Our data reveal a delayed time of introduction of CFs in children with ASD. The age of CFs introduction is associated with later feeding problems. ASD subjects present more feeding problems than TD subjects, which are clearly associated with symptoms of ASD. Based on the collective findings, we propose that clinicians should pay attention to infants presenting with difficulties in adjusting to introduction of dietary CFs who may have more feeding problems later and give the parents some guidance.

## Data availability statement

The raw data supporting the conclusions of this article will be made available by the authors, without undue reservation.

## Ethics statement

The studies involving human participants were reviewed and approved by the Ethics Committee of the First Hospital of Jilin University. The parents of participants provided written informed consent to participate in this study.

## Author contributions

XY and TW devised the project and the main conceptual ideas, worked out almost all of the technical details, and performed statistical analyses with assistance from FJ. XY, TW, LS, YX, and JF performed data collections and measurements. TW wrote the manuscript, with assistance from XY. All authors contributed to the article and approved the submitted version.

## Funding

The study was supported by the National Nature Science Foundation of China (81973054), Key Scientific and Technological Projects of Guangdong Province (2018B030335001), Effect of vitamin D regulating glutamate NMDA receptor on symptoms of autism rat model and its mechanism (20200201507JC).

## Conflict of interest

The authors declare that the research was conducted in the absence of any commercial or financial relationships that could be construed as a potential conflict of interest.

## Publisher's note

All claims expressed in this article are solely those of the authors and do not necessarily represent those of their affiliated organizations, or those of the publisher, the editors and the reviewers. Any product that may be evaluated in this article, or claim that may be made by its manufacturer, is not guaranteed or endorsed by the publisher.
